# Neurologic Deterioration in Acute Traumatic Central Cord Syndrome Without Bone Injury Caused by Traumatic Cerebrospinal Fluid Rhinorrhea: A Case Report

**DOI:** 10.7759/cureus.93072

**Published:** 2025-09-23

**Authors:** Michiru Katayama, Yasufumi Ohtake, Fukuda Mamoru, Yuma Hiratsuka

**Affiliations:** 1 Neurological Surgery, Nakamura Memorial Hospital, Sapporo, JPN

**Keywords:** acute traumatic central cord syndrome, neurologic deterioration, skull base fracture, surgical treatment, traumatic cerebrospinal fluid rhinorrhea

## Abstract

Whether to perform surgery for acute traumatic central cord syndrome (ATCCS) without bone fracture or dislocation but with spinal cord compression due to preexisting cervical canal stenosis remains controversial. We present a case of ATCCS without bone injury in which neurologic deterioration was possibly caused by traumatic cerebrospinal fluid rhinorrhea (TCFR). An 82-year-old man with ATCCS (American Spinal Injury Association (ASIA) Impairment Scale (AIS) C) and an anterior skull base fracture was urgently transported to our hospital and was initially treated conservatively. However, on the day after admission, his quadriparesis worsened, and MRI showed high signal intensity in the epidural area on both T1-weighted and T2-weighted images, initially suggesting an epidural hematoma. In response to this neurological deterioration, we decided to perform urgent surgery aimed at spinal cord decompression. Although there was no obvious TCFR at admission, profuse TCFR was observed upon prone positioning for surgery. A lumbar CSF drain was inserted to control the CSF leak, but output remained negligible, consistent with marked intracranial hypotension. C3 laminectomy and C4-C6 laminoplasty were performed with the patient in the head-elevated position, leading to neurological improvement. No epidural hematoma was found intraoperatively. The abnormal MRI findings were presumed to be due to engorgement of the epidural venous plexus associated with low CSF pressure. Subdural hygroma was noted on head CT on postoperative day 7, which resolved spontaneously by day 14. To the best of our knowledge, there have been no previous reports describing exacerbation of cervical spinal cord compression due to intracranial CSF leakage in the setting of preexisting cervical stenosis. This report suggests that in patients with ATCCS and concomitant anterior skull base fracture, TCFR may contribute to neurological deterioration. Further accumulation of such cases is required to clarify this association.

## Introduction

Acute traumatic central cord syndrome (ATCCS) is the most common form of incomplete spinal cord injury (SCI) [1]. For ATCCS without bone injury, the indication for surgery and the optimal timing remain controversial [2-7]. Recently, reports recommending early surgery within 24 hours after injury have increased; however, evidence regarding its superiority over conservative treatment remains limited [2,3,6]. Consequently, in Japan, even at present, elderly ATCCS patients in poor general condition are often treated conservatively [3-6].

While most ATCCS cases improve with conservative treatment, neurologic deterioration can occur [8-10]. There are a few articles that discuss the mechanisms by which ATCCS worsens during conservative treatment [11]. Furthermore, the evidence supporting surgery when neurological symptoms worsen is unclear, and decisions are currently left to the treating physician.

In this report, we describe a case in which TCFR was presumed to have contributed to the neurological deterioration of ATCCS during conservative management. In this case, urgent spinal cord decompression resulted in neurological improvement. To the best of our knowledge, there have been no previous reports describing exacerbation of cervical spinal cord compression due to intracranial CSF leakage in the setting of preexisting cervical stenosis. However, cases of rapid neurological deterioration following lumbar puncture in patients with spinal canal stenosis have been reported [12,13]. Proposed pathophysiological mechanisms include spinal coning due to sudden changes in the CSF pressure gradient, increased spinal cord compression from engorgement of the epidural venous plexus under low CSF pressure, and exacerbation of direct spinal cord compression due to loss of the buffering effect of CSF [12,13]. We believe similar mechanisms may have been involved in the present case. 

## Case presentation

The patient was an 82-year-old man with a history of myocardial infarction and chronic kidney disease. After undergoing dialysis, he became unsteady at home and fell forward, injuring his face. He presented with tetraparesis and was rushed to our hospital. On physical examination, he was alert with stable vital signs. There were periorbital ecchymosis and edema, and he had severe pain, numbness, and allodynia predominantly in the upper extremities.

**Figure 1:**
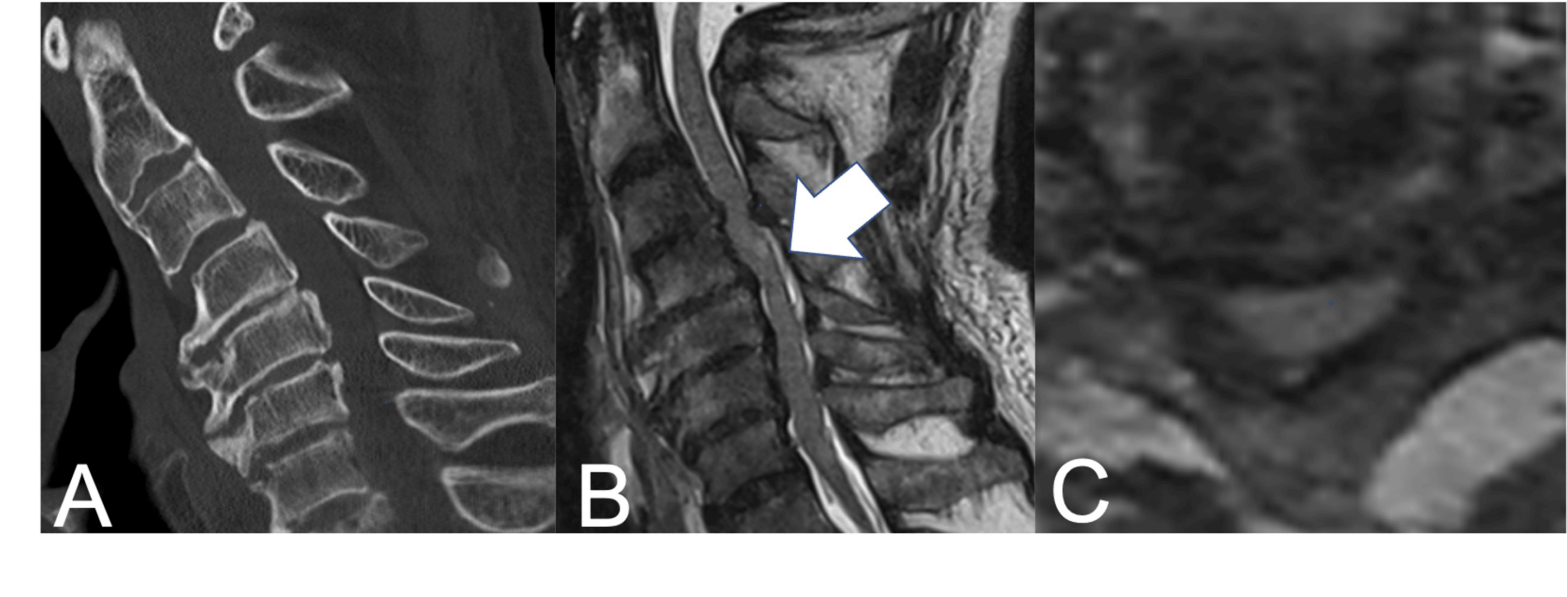
(A) Sagittal CT scan at admission show no bone injury; (B) Sagittal T2-weighted MRI on admission indicate mild intramedullary signal change at the C3/4 level (white arrow); (C) Axial T2-weighted MRI at admission show severe cervical spinal canal stenosis.

When assessing motor strength using the International Standards for Neurological Classification of Spinal Cord Injury (ISNCSCI), there was an 11-point difference in manual muscle testing (MMT) between the upper (15 points) and lower extremities (26 points), indicating ATCCS of American Spinal Injury Association (ASIA) Impairment Scale (AIS) C. Cervical spine CT showed no bone injury, and MRI revealed spinal canal stenosis and mild intramedullary signal change at the C3/4 level (Figure 1).

**Figure 2:**
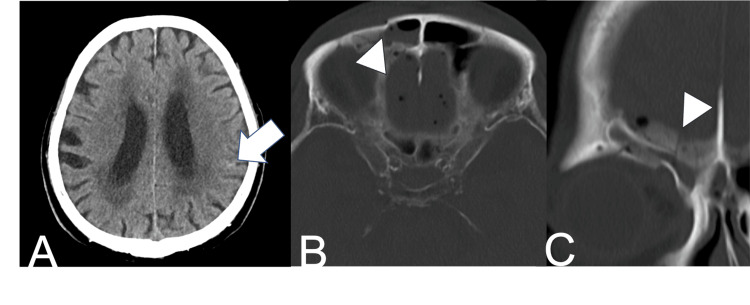
(A) Axial head CT scan at admission show mild traumatic subarachnoid hemorrhage (white arrow); Axial (B) and coronal (C) head CT scans at admission show anterior skull base fracture (arrowheads) and pneumocephalus.

Head CT showed mild traumatic subarachnoid hemorrhage, anterior skull base fracture, and pneumocephalus (Figure 2).

No obvious CSF leak was detected at admission. We initially decided to treat both the head injury and the cervical spine condition conservatively. The patient was kept at bed rest with the head in a horizontal position. External immobilization with a cervical collar was applied, and analgesics were started.

**Figure 3:**
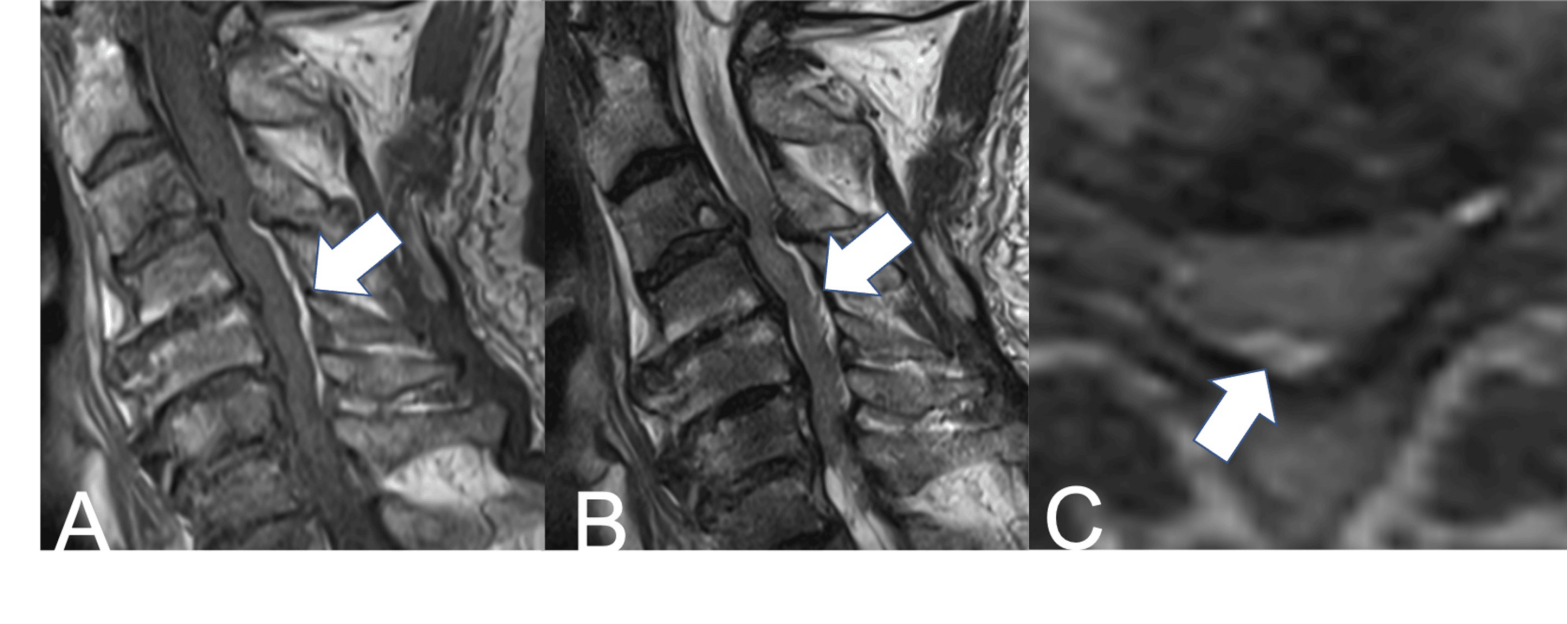
(A) Sagittal T1-weighted MRI at the time of deterioration; (B) Sagittal T2-weighted MRI at the time of deterioration; (C) Axial T2-weighted MRI at the time of deterioration Arrows show high signal intensity in the epidural area on both T1-weighted and T2-weighted images.

However, on the day after admission, when the patient’s muscle strength was re-evaluated with ISNCSCI, the MMT score of the upper extremities had deteriorated to 10 points and the lower to 15 points. MRI showed no worsening of intramedullary edema, but high signal intensity was observed in the epidural area on both T1-weighted and T2-weighted images (Figure 3).

**Figure 4:**
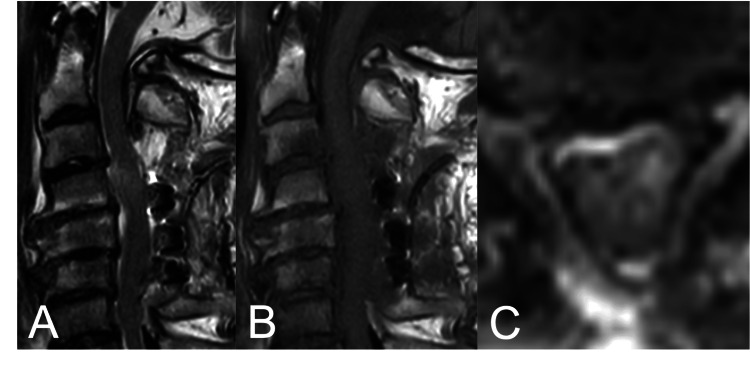
Laminectomy at C3 and laminoplasty from C4 to C6

Initially, an epidural hematoma was suspected. Given neurologic deterioration despite conservative management, we decided to perform emergency laminoplasty. When the patient was placed in the prone position, continuous and profuse CSF rhinorrhea was observed. To control CSF leakage, a lumbar CSF drain was inserted, but the drain output remained negligible, and surgery was performed with the head elevated. No epidural hematoma was identified intraoperatively, and the abnormal MRI signal observed preoperatively was presumed to represent dilation of the epidural venous plexus due to the CSF rhinorrhea. The surgery was completed without complication (Figure 4).

Fortunately, following surgical treatment, evaluation with ISNCSCI on the next day showed that the MMT score had improved to 20 points in the upper extremities and 30 points in the lower extremities. Because the spinal drainage produced minimal output throughout and was considered likely to worsen intracranial hypotension, it was removed on the day after surgery.

**Figure 5:**
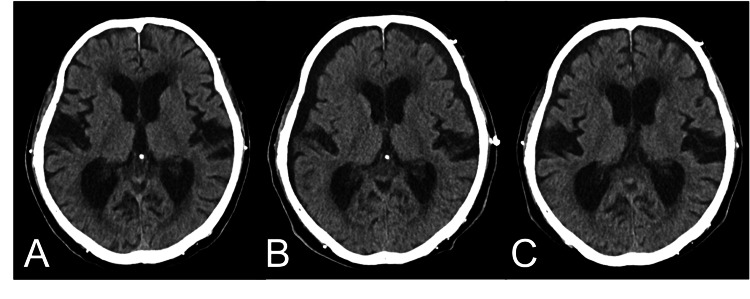
Axial head CT scan at (A) day 1 show no evidence of intracranial hypotension, (B) day 7 reveal subdural hygroma and narrow ventricular, suggesting intracranial hypotension, and (C) day 14 show improvement.

For the TCFR, conservative treatment with head elevation was performed. Head CT on day 7 revealed findings of subdural hygroma, which subsequently resolved spontaneously on day 14 (Figure 5).

**Figure 6:**
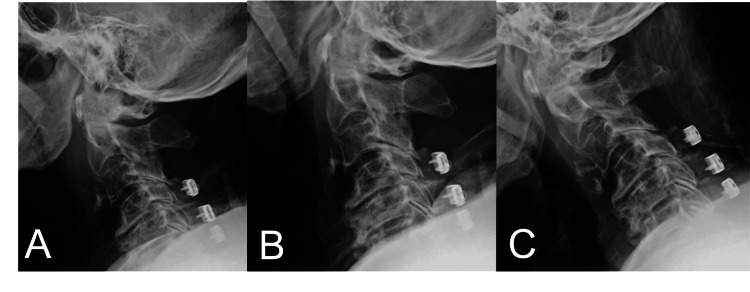
Dynamic radiographs taken one month after surgery show no instability of the cervical spine.

Dynamic radiographs taken one month after surgery showed no instability of the cervical spine (Figure 6). However, the patient developed a urinary tract infection and died two months after surgery.

## Discussion

SCI is a devastating condition that results in lifelong disability, long-term risks of medical complications, and substantial use of health care resources. Previously, SCIs were recognized as a condition that primarily affected young people, often caused by motor vehicle collisions and other high-velocity mechanisms [1]. However, in Japan, an aging society, most incomplete traumatic SCIs in older adults are ATCCS without fractures [6]. Schneider defined ATCCS as “a syndrome of acute central cervical SCI characterized by disproportionately more impairment of the upper than the lower extremities, bladder dysfunction, usually urinary retention, and varying degrees of sensory loss below the level of the lesion” [14]. Although patients may experience only minor or moderate trauma, ATCCS is characterized by heterogeneity in symptoms and severity. Regarding this diverse pathology, the choice between surgical and conservative treatment remains controversial [2-7].

The causes of ATCCS can be categorized into four pathoanatomic contexts: (i) MRI evidence of intramedullary signal change without other radiographic abnormality, (ii) acute disc herniation, (iii) fracture-subluxation injuries, and (iv) spinal stenosis without bony or ligamentous injury. Surgery is generally accepted for ATCCS secondary to acute disc herniation, fracture, and/or instability. There is also a consensus that surgical treatment is not indicated for non-stenotic ATCCS, which primarily occurs in young people [2]. However, for ATCCS with preexisting stenosis, which often occurs in the elderly, both the indication for surgery and the timing remain controversial [2-7].

There is significant heterogeneity in diagnosis [7], and ATCCS often improves spontaneously. Additional benefit beyond natural recovery must therefore be demonstrated to justify surgery, but the evidence remains insufficient [7]. Two prospective studies conducted in Japan found that surgery was no more effective than conservative treatment in improving paralysis in patients with ATCCS [4,5]. A multicenter, retrospective study conducted by Nori et al. found that there were no significant differences between surgical and conservative treatment groups, regardless of surgical timing, in terms of AIS grade, ASIA motor score at six months after injury, or change in ASIA motor score from baseline to six months [6]. In contrast, Nakajima et al. reported that surgical treatment should be recommended to restore walking ability in patients with spinal cord compression of ≥33.2% [15].

The timing of surgery is also controversial. In recent years, reports recommending early surgery within 24 hours after injury have increased; however, evidence regarding its superiority over conservative treatment remains limited [2,3,6]. The AO Spine-led multidisciplinary guideline development group weakly recommended early decompression for patients with acute central cord syndrome, given its potential benefits on neurological and functional outcomes [3]. By contrast, a randomized clinical trial in AIS C patients with cervical SCI without major bone injuries found that early surgical treatment (<24 hours) accelerated recovery within the first six months but had similar effects as delayed surgical treatment (>2 weeks) at one year after injury [16]. From this perspective, in the current case, considering that the patient was at high risk for emergent surgery (advanced age, hemodialysis, and a history of angina), we initially chose conservative treatment.

Unfortunately, the day after admission, his neurological symptoms worsened. While most ATCCS cases improve with conservative treatment, neurologic deterioration can occur [8-10]. Evidence to support surgery when neurological symptoms worsen remains limited. Jin et al. reported in 17 operative cases that timely spinal decompression after recurrent neurologic deterioration resulted in good neurological outcomes [11]. On the other hand, Katoh et al. reported that five of 63 patients experienced neurologic deterioration, and all but one patient recovered without surgery [17]. Currently, it is up to each physician to decide whether to perform surgery or continue conservative treatment.

To date, several reports have discussed factors that worsen cervical SCI. Farmer et al. reported correlations with sepsis, dehydration, intubation, and ankylosing spondylitis [8]. Harrop et al. categorized the onset of neurologic deterioration into three temporal subsets [10]: early (<24 hours), typically related to traction and immobilization; delayed (24 hours to seven days), associated with sustained hypotension in patients with fracture-dislocations; and late (>7 days), observed in a patient with vertebral artery injuries. However, there are a few reports that discuss mechanisms of deterioration in ATCCS without bone injury. Jin et al. reported that, even in the absence of fracture, ruptures of the anterior longitudinal ligament (ALL), posterior longitudinal ligament (PLL), and discs resulting in cervical instability were important causes of recurrent neurologic deterioration after conservative treatment for ATCCS [11]. In our case, dynamic radiographs taken one month after surgery showed no cervical instability. Notably, in that series, the median time to deterioration was 50 days, and the shortest was 10 days, suggesting that other mechanisms may operate during the acute phase.

Based on our experience with this case, we suggest that TCFR may be one of the contributing factors to the deterioration of ATCCS. We found no prior reports describing worsening of cervical spinal cord compression due to intracranial CSF leakage in the presence of preexisting cervical canal stenosis. By contrast, several cases have been reported in which patients with spinal canal stenosis experienced acute neurological deterioration following lumbar puncture [12,13]. Proposed mechanisms include spinal coning due to abrupt CSF pressure gradients, epidural venous plexus engorgement under low CSF pressure, and loss of the CSF buffering effect [12,13].

In the present case, intraoperative insertion of a lumbar CSF drain yielded minimal output, and postoperative head CT revealed a subdural hygroma. These findings are consistent with intracranial and intraspinal hypotension, and we believe the neurological deterioration occurred through a mechanism similar to those previously described in post-lumbar puncture cases [12,13]. Additionally, an MRI performed at the time of neurological deterioration demonstrated a dorsal epidural lesion with high signal intensity on both T1- and T2-weighted images. Although an epidural hematoma was initially suspected, no hematoma was identified intraoperatively. Therefore, we considered that the abnormal MRI signal was more likely attributable to dilation of the epidural venous plexus associated with low CSF pressure. Previous reports have documented cases in which dilated epidural venous plexus under low CSF pressure contributed to worsening of myelopathy or radiculopathy [18,19]. The Monro-Kellie hypothesis-that the sum of intracranial brain, CSF, and blood volumes is constant-likely underlies this pathophysiology; an increase in one component should cause a decrease in one or both of the remaining two [20]. Thus, when intracranial CSF decreases, venous volume increases to compensate, with engorgement extending to the spinal epidural venous plexus. When such venous engorgement is superimposed on spinal canal stenosis, venous drainage may be further compromised, potentially resulting in spinal cord swelling and exacerbation of compression [18-20].

Taken together, TCFR may exacerbate ATCCS. In light of the presumed pathophysiology, timely spinal cord decompression may represent an effective therapeutic option. Patients with SCI accompanied by skull base fracture warrant careful management, as CSF leakage may precipitate neurological deterioration. Finally, because this is a single-case report with a short follow-up period, further validation through the accumulation of additional cases is essential.

## Conclusions

This report describes a single case in which TCFR was suspected to have worsened preexisting cervical canal stenosis and contributed to neurological deterioration in ATCCS. So patients with SCI accompanied by skull base fracture warrant careful management.
